# Medical student´s motivational changes during the COVID-19 university lockdown: a mixed-method study

**DOI:** 10.1186/s12909-024-05204-9

**Published:** 2024-03-04

**Authors:** Parisa Moll-Khosrawi, Josephine Küllmei, Viorel Chindris, Alexander Ganzhorn, Jan Marcus Haus, Christian Zöllner, Leonie Schulte-Uentrop

**Affiliations:** Department of Anaesthesiology, University Medical Centre Hamburg-Eppendorf, Martinistr. 52, 20246 Hamburg, Germany

**Keywords:** Motivation, Face-to-face teaching, Remote teaching, Patient contact

## Abstract

**Background:**

During the crucial stage of the COVID-19 pandemic, face-to-face undergraduate medical education was disrupted and replaced with online teaching activities. Based on its emphasized impact on several outcomes, a deeper insight into the pandemic related effects on medical students´ motivation is aspirational. Therefore, this study aimed to assess the motivational changes that took place during the pandemic in medical students and explored, how motivation of medical students is influenced.

**Methods:**

Using a mixed method inter-cohort study design, 4th year medical students´ motivation, assessed pre- and post-pandemic were compared. In subsequent qualitative analyses underlying variables that may have contributed to both- medical students´ motivation and pandemic related changes were identified. These variables were then systematically explored- both individually and in combination. In a final step, the results were embedded within the Self-Determination Theory.

**Results:**

Students who were affected by the university lockdown reported significantly higher levels of less self-determined motivation and amotivation. The qualitative analysis identified determinants that influence medical students´ motivation. The common core of these determinants is lacking social interaction and support, with a great emphasis on the interaction with the lecturer and patients.

**Conclusion:**

This study emphasizes the crucial role of medical educators, patient contact, social interactions and personal support on students´ motivation. Students need to be strengthened in their beliefs about their abilities, the value of their task at hand and receive encouragement in their efforts. All this will result in an increased identification with the task and less detrimental outcomes.

## Background

During the crucial stage of the COVID-19 pandemic, social distancing became necessary to flatten off the number of new COVID-19 cases. Therefore, face-to-face undergraduate medical education was disrupted in many countries and replaced with online teaching activities [1].

The implementation of effective COVID-19 vaccination and decreasing numbers of COVID-19 cases [2] were promising prospects to end the pandemic and return to pre-pandemic circumstances in many aspects of daily life, including teaching at universities [3]. Many universities relaunched their face-to-face teachings at winter semester 2021/22. Likewise, at Hamburg medical school courses started up again to their full extent at winter semester 2021/22 moving from three semesters of solely online teaching to face-to-face education.

During the university lockdowns, research has been conducted on how online teachings of medical curricula could be optimized, predominantly with the goal to ensure effective teaching and to prevent a decrease of medical students´ motivation [4–6]. None of these studies targeted to provide a deeper insight in the motivational dimension. However, studying students´ motivation is important, as according to learning psychology, the motivational dimension of learning is equal to the cognitive and metacognitive dimensions [7].

The commonly applied theory to study motivation is the “Self Determination Theory” (SDT) [8]. SDT postulates that the satisfaction of three basic psychological needs- autonomy, competence and relatedness- determines the personal growth of every human being [9]. The satisfaction of these needs results in different types of motivation that underlie human behavior [10]. The behavioral regulations define where the locus of causality, meaning the reason to engage in an activity, is perceived to lie in [8]. The most internal perception results in intrinsic behavioral regulation and- in decreasing order- moving more towards external perception in the following regulations: identified-, introjected-, external regulation [10]. External and introjected regulation are summarized as “controlled self-regulation” and intrinsic and identified regulation are summarized as “autonomous self-regulation”. Amotivation signifies that the individual has no motivational quality and furthermore has been linked to detrimental effects (a.e depression) in medical students [11]. Autonomous regulation is associated with several positive outcomes (a.e. better learning, better academic achievement, better well-being, perseverance and enthusiasm), whereby controlled regulation has been linked to more negative outcomes in medical students [12–15].

Little is known about other variables than teaching methods [16], affecting medical students´ motivation. A greater insight into the motivational dimension of learning is aspirational, as this dimension has been poorly understood and neglected in medical education [17]. Furthermore, assessing the pandemic related effects on medical students´ motivation is crucial to evaluate the necessity of interventions and curriculum amendments to circumvent possible adverse effects of the motivational changes.

We therefore aimed to assess the motivational changes that took place during the pandemic in medical students and systemically explored how motivation of medical students is influenced. In a mixed method inter-cohort study design, first the situational motivation to engage in classes was assessed within two cohorts of 4th year undergraduates. After the relaunch of face-to-face teachings in medical school, the situational motivation was assessed within one cohort and compared to the motivation reported by 4th year students prior to the pandemic (quantitative measure). In subsequent qualitative analyses, underlying variables that may have contributed to both- medical students´ motivation and pandemic related changes were identified. These variables were then systematically explored- individually and in combination. Furthermore, to work out specific knowledge and implications for medical education, the findings were embedded in the context of the SDT.

We hypothesized that after the lockdown, the students would report higher levels of autonomous (intrinsic, identified) motivation towards participating in the same face-to-face teaching units (primary endpoint), than reported by students prior to the university lockdown.

## Methods

### Study setting

This study was performed at the Department of Anaesthesia of the University Medical Center Hamburg Eppendorf, during the winter semester 2021/22. Additional data was included in the analysis, collected during the winter semester 2019/2020 in the context of a study which aimed to assess the course of motivation during medical school and had to be terminated due to the pandemic.

At Hamburg University Medical School, the anaesthesiology curriculum is organized as a learning spiral [18]. After passing the introductory classes (Basic Life Support in 1st year and an introduction to Advanced Cardiac Life Support in their 2nd year), the undergraduates attend Advanced Cardiac Life Support II in their 3rd year- and Advanced Cardiac Life Support III in their 4th year of medical school. These teaching units are compulsory and are composed of a seminar and subsequent simulation training.

As each training has a limited capacity, the teaching units are scheduled throughout the whole semester and the undergraduates are aligned through the Deans´ office.

Prior to the pandemic, the teaching units of the department of anaesthesiology have been very popular at Hamburg University Medical School which was also reflected in the students’ evaluation. According to pandemic regulations, the medical curriculum of the University of Hamburg was modified into e-learning based teaching (starting summer semester 2020). All teaching units were recorded and asynchronously made available on a learning platform. Additionally, the lessons were held synchronously online and were broadcasted via *Cisco Webex™ Online Meetings, Milpitas, California, US.* During the study period, face-to-face teachings were relaunched after three semesters of online teaching.

### Study design

A mixed method inter-cohort study design which included quantitative assessments and focus groups (qualitative analysis) was applied.

To analyze the effects of the pandemic on students´ motivation, 4th year students´ motivation was assessed after the relaunch of universities and then compared to those of pre-pandemic 4th year medical students.

For this quantitative assessment data was collected using the German “Situational Motivation Scale” (SIMS) [19], which has been validated in preceding investigations [20, 21]. 

The qualitative analysis included three subsequent in-depth-interviews (focus groups) with different participants, as well as expert group analysis. Focus groups have have gained a broad application in medical education [22]. This method is appropriate to detect the reason why something is (not) observed- targeting the nature of phenomena [23].

A threefold analytic strategy was used: Data from the quantitative analysis (data-driven) displayed the current state, which was explained by the qualitative analysis (explanation-driven) and was then embedded within the leading theory of motivation, SDT (theory-driven).

**Fig. 1:**
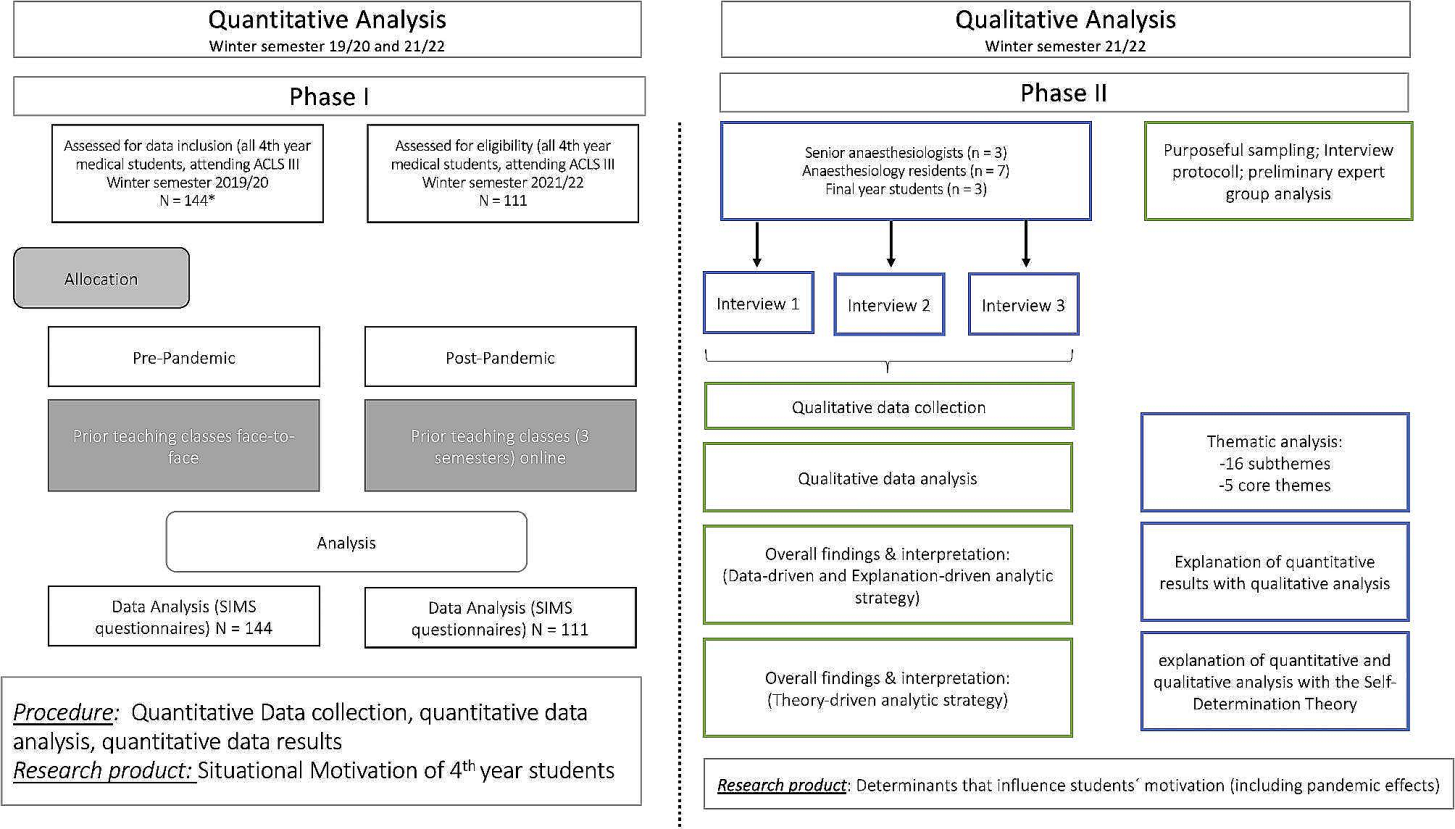
Process flow diagram of the procedures and participant flow. Note: Depicted is the study procedure with the qualitative and quantitative analysis; the green framed squares describe the process and the blue framed squares describe the corresponding procedure of the qualitative part. Abbreviations: ACLS = Advanced Cardiac Life Support; SIMS = Situational Motivation Scale. * Data collected during a prior study which was interrupted due to the pandemic

An overview of the study design and participant flow is provided in Fig. 1.

### Quantitative analysis

#### Participants

All 4th year undergraduates who were aligned to attend the mandatory anaesthesiology module (ACLS III) during the winter semester 2021/22, were eligible. During the post-pandemic semester, students who had symptoms of illness or were tested positive for the COVID-19 virus were excluded. The undergraduates were informed about the study prior to their training. Participation in study was voluntary and written informed consent was collected from each study participant. To prevent potential bias due to outer circumstances (other than the pandemic), the cohorts were under similar conditions (a.e comparison of winter semester).

#### Outcome of the quantitative analysis

Situational motivation of the undergraduates towards participating in the face-to-face teaching unit and studying medicine was assessed using a translated version of the “Situational Motivation Scale”, adapted by Gillet et al. [19, 20]. The students in bothy study groups (pre- and post-pandemic) were provided with the SIMS questionnaire at the beginning of their class- prior to the actual teaching unit- and were explicitly asked to answer the items of the SIMS with regard of why they study medicine.

The SIMS measures the type of motivation to engage in an activity at a specific point of time, [19] exploring why an individual shows a certain behaviour [24]. It is possible to compare the assessed motivation with its conceptual definition that expels to the recognized reason of task engagement and is therefore appropriate to compare students´ motivation at different points of time as well as the development of motivation during a period [9, 25].

The adapted version of the SIMS measures intrinsic motivation, extrinsic-, identified-, introjected regulation and amotivation on of five subscales. Each subscale is composed of four items and each item has a 7- point Likert scale (1 = “Does not correspond at all” and 7 = “Corresponds exactly”). The motivational qualities (subscale) are computed based on the corresponding items with four items per subscale [19]. No specific cut-off values for the subscales of the SIMS have been reported to characterize or categorize the level of the motivational qualities. However, the scores can be interpreted considering the differences.

By adding and averaging the intrinsic motivation and identified regulation, an autonomous motivation index can be computed. Similarly, a controlled motivation index can be computed by adding and averaging extrinsic- and introjected motivation [19, 26].

The validity and reliability of the SIMS, as well as of the adapted and translated version, have been confirmed in several studies [19, 21, 27].

### Qualitative analysis

#### Participants

Participants of the qualitative analysis were selected purposefully. The participants of the focus groups were invited by the leader of the research group. Senior anaesthesiologists who were actively involved in medical education (*n* = 3), anaesthesiology residents- also involved in medical education (*n* = 7) and final year undergraduates (*n* = 3) were selected. The participants were not familiar with the concept of SDT. The expert group was composed of three anaesthesiologists who are actively involved in the coordination of medical education at the department of Anaesthesiology.

#### Outcomes and procedure of the qualitative analysis

Before the outcomes of the quantitative analysis were assessed, the authors initially drafted an interview protocol targeting the research questions. The protocol was piloted and then revised based on question-answer clarification and feedback. Then the interviews were conducted by the authors until saturation (no further data were needed based on agreement on the topics of interest) [28]. To prevent potential bias, the leader of the research group- who was familiar with the concept of SDT- did not conduct the interviews. A triangulation strategy was applied to assess data validity [29]. Further critical evaluation of the findings (data rigor) was conducted regarding reliability and objectivity, credibility, transferability, trustworthiness, and confirmability [29]. The interviews were recorded and then transcribed. Next to the recordings, notes were taken during the interviews and the relevant statements of each interview were summarised graphically by each group in sense of prolonged engagement with data (validity; confirmability; credibility) [30]. The leader of the research group and another author listened and analysed the interviews multiple times; obscurities were resolved by checking the transcripts and recordings of the interviews (preliminary analysis). A second member of the research group repeated the same process independently (validity). The analysis was presented to members of the research group for further confirmation. Data was then analysed by an independent group of staff members (peer checking; dependability; consistency) [31]. Subsequently, data was analysed by an expert group using the mapping and template method, as well as direct conduct analysis as described by Graneheim and Lundman [32–34]. In a sequential and inductive process relevant data content was condensed systematically by highlighting answers and statements that were directly linked to the research question. Important statements were coded and similar codes were gathered to themes and subthemes. The results were then explained by the self-determination theory. This process was conducted until saturation was reached [28]. Three external and independent members of the faculty, with comparable characteristics of the participants, reviewed the findings and confirmed the consistency of the results (dependability; consistency; transferability) [31, 35].

Each focus group interview began with the purpose of the study and with a short description how the in-depth interviewing will be conducted. As the interviewing was semi-structured, the order of the questions was not fixed. The interviews were composed of two parts, in the first section the results of the quantitative assessment were not displayed.

The interview protocols were drafted based on the aims of the study and targeted to collect and explore:

1. Experiences and perspectives on medical students´ motivation and its alterations.
2. General factors influencing medical students´ motivation.
3. Effects of the pandemic and the forced disruption of face-to-face teachings on medical students´ motivation.

In the second part of the interview, the results of the motivational changes, assessed in the quantitative analysis, were displayed and considered. The variables (student motivation and pandemic effects) were then systematically explored- individually and in combination.

### Statistical analysis

Statistical analysis was performed with SPSS (version 28.0.1.0, IBM Corp., Armonk, New York, USA). Descriptive statistics (mean values, standard deviations) were calculated for all data.

There were no to minor outliers according to inspection with a boxplot. Data was not normally distributed for each teaching unit (Shapiro-Wilk test, *p* < 0.05). Homogeneity of variances was not asserted (Levene´s Test, *p* < 0.05), therefore, Welch´s t-test was conducted to compare the motivational qualities for the teaching units prior the pandemic and after the relaunch of face-to-face teachings (post-pandemic). A two-tailed *p* < 0.05 was considered to be statistically significant.

## Results

### Participants and SIMS questionnaires

A total of *n* = 111 undergraduates were eligible in the post-pandemic semester and participated in the study. Data of *n* = 144 pre-pandemic students was included. A total of *N* = 255 SIMS questionnaires were collected. All questionnaires were complete and included within the analysis.

### Quantitative outcome measures

Overall, the undergraduates of both cohorts reported high levels of autonomous motivation (intrinsic-, identified regulation) and low levels for controlled motivation (extrinsic-, introjected regulation). The reported levels of amotivation were also low (Table 1).

**Table 1:** Motivational levels reported by 4th year undergraduates under pre- and post-pandemic circumstances

	Pre-pandemic		Post-pandemic				95% CI	
Motivational Quality	MV	SD	MV	SD	T(df)	p	LL	UL
*Intrinsic* *Identified* *Introjected* *Extrinsic* *Amotivation*	5.17 5.73 3.32 3.40 1.29	1.16 0.88 1.24 1.39 0.52	5.13 5.60 3.52 4.03 1.61	1.22 1.20 1.59 1.73 0.83	0.26 (230.55) 0.94 (194.39) -1.08 (203.25) -3.16 (207.21) -3.61 (172.67)	0.798 0.248 0.281 0.002* < 0.001*	-0.258 -0.139 -0.559 -1.028 -0.505	0.345 0.349 0.163 -0.237 -0.148
*Motivation* *indices*								
*Controlled* *Autonomous*	3.36 5.45	1.10 0.92	3.77 5.37	1.42 1.13	-2.54 (201.96) 0.64 (253)	0.009* 0.531	-0.738 -0.176	-0.103 0.343

Note: *p < 0.05. Abbreviations: CI = confidence interval; LL = Lower limit; UL = Upper limit; MV = Mean value; SD = Standard deviation

There were significant differences in extrinsic regulation and amotivation between the cohorts participating in the pre-pandemic- and post-pandemic teaching units. Students who participated in the teaching units after the university lockdown reported 0.63 points (95%-CI[-1.03, -0.24]) higher levels (measured on the SIMS scale) of extrinsic regulation, *t*(207.21)= -3.16, *p* = 0.002 than students who participated in the teaching units prior to the pandemic. The levels of amotivation reported by the post-pandemic cohort of students was also significantly higher with a mean difference of 3.27 (95%-CI[-0.50, -0.15]) points, *t*(172.7)= -3.61, *p* < 0.001. The index of controlled regulation, which is computed by adding and averaging extrinsic- and introjected regulation was therefore also significantly higher with a mean difference of 0.42 (95%-CI[-0.74, -0.09]) points, *t*(201.96)= -2.54, *p* < 0.012.

Our results did not confirm our hypothesis, that after the lockdown, students report higher levels of autonomous motivation towards participating in the teaching classes, compared to the cohort of pre-pandemic students. In contrast, our results indicate that within the cohort of students who attended the teaching units after the lockdown, extrinsic levels of motivation, controlled regulation and amotivation had significantly grown.

### Qualitative analysis

A total of sixteen subthemes were identified and classified under five core themes, as displayed in Table 2. These core themes were composed of the following: Interaction with the lecturer; disruption of face-to-face teaching units; disruption of patient contact; disruption of daily university life structure; social (peer) isolation through closure of universities.

### Theme 1: interaction with the lecturer

The impact of the interaction with the lecturer, identified by the interview participants, is discussed under three subthemes: *Endorsement of competence through feedback; monitoring of the learning cycle through feedback; the lecturer as a role model*. The participants mainly stated that the lack of contact with the lecturer led to decreased identification with being a doctor, as the lecturer is seen as a role model. Even the negatively perceived lecturers endorse the identification process, by rising feelings of refusal and simultaneous enhancement of the awareness of what students want to become. The participants stated that the contact with the lecturer monitors the learning cycle and progress, which was not sufficient during online classes. Overall, the missing contact with the lecturer led to feelings of incompetence (lack of feedback and monitoring of learning) and decreased the feeling of identification with being a doctor.

### Theme 2: disruption of face-to-face teaching units

Participants identified some challenges resulting from the disruption of face-to-face teaching units. Although the lecturers were eager to provide feedback during the web-based teaching classes and tried to conduct the classes interactively, the identification of knowledge gaps and the opportunity to fill these gaps (usually provided in the face-to-face teachings) were clearly missing. The positive confirmation of what students had already learned was lacking due to many factors during online classes. Furthermore, the connections of the learning contents were not clearly seen, the learning content was not structured like in face-to-face teachings. All together leading to cognitive overload. In summary the feeling of incompetence grows due to the disruption of face-to-face teachings.

### Theme 3: disruption of patient contact

The impact of patient contact on students´ motivation was emphasized greatly by the participants. One of the main obstacles of the restricted access to clinical environment was stated to be the disrupted patient contact. Contact with patients enhances the feeling of wanting to become a doctor and to put effort into achieving this goal. The feeling of wanting to help is nourished- and hereby the identification with the process of becoming- and also the identification with being- a doctor is fostered. The contact with patients highlights the importance and the value of studying and hereby facilitates this process. Participants stated that feelings of competence and autonomy grow through patient contact, mainly because students get a sense of their future role and also because students can try out their knowledge (of course under supervision).

### Theme 4: disruption of daily university life structure

The participants stated that the structure of university is a reliable constant of daily life and provides a feeling of security. Even when inconsistencies occur in daily life- the structure provided by university provides something to rely on and structures daily life. The restricted access to university created a sense of insecurity, overload and helplessness (being all alone with structuring studying). Participants also stated that going to university is a valuable experience, creating a sense of freedom. Furthermore, the reason to study is seen (figuratively speaking) and hereby valued.

### Theme 5: social (peer) isolation through closure of universities

Participants pointed out the immense effect of peer-interaction on students emotional and motivational state. Through interaction knowledge and experiences are shared, feelings of competence fostered and the meaning and value of studying highlighted. The feeling of being a medical student is reinforced multilaterally. Positive social pressure was lacking and hereby learning devalued. Feelings of insecurity, being alone, incompetence and alienation grew.

**Table 2:** Summary of themes and sub-themes and analysis according to SDT

Theme	Sub-theme (description)	Pandemic consequences related to SDT
*1. Interaction with the lecturer* Interaction with the lecturer as a process of enhancing the feeling of competence, identification with being a doctor and facilitating the learning cycle Interaction with the lecturer	1.1 *Endorsement of* *competence through feedback* Interaction with the lecturer and its feedback endorse the feeling of competence 1.2 *Monitoring of the* *learning cycle through feedback* The learning process is monitored through the interaction (feedback) with the lecturer *1.3 The lecturer is a role model*	Through reduced contact (only possible via email or web-based lessons), the function of the lecturer as a role model was decreased. Hereby the identification with being a doctor was also decreased and the internalization of studying to become a doctor was also reduced. The result is a shift towards external regulated behavior. The lack of feedback and monitoring of the learning cycle (due to web-based lessons) resulted in a decreased feeling of competence with an accompanied decrease of ability beliefs- all nourishing amotivation. The ability beliefs of a student are influenced at most by the teacher and its competence support. The lecturer (role model) functions as a key figure for medical students. The need for interpersonal affiliation develops when good relationships with these key figures develop. Students need stable relationship with these key figures, they are nurturing for internalization and hence autonomy supportive in terms of motivation, increasing relatedness and decreasing amotivation. Due to the pandemic these relationships were reduced and therefore relatedness to those key figures vanished and amotivation increased.
***Sample statements for theme 1.***	*“In the teaching units the educators always gave us feedback and that helped me to organize the learning at home and supported me to identify the learning contents which I should focus more on.” (S2)* *“I felt like a part of a whole and mostly I wanted to become like our educators.” (S3)*	*“At the beginning of each class, we usually display the learning goals, and we check regularly if they have been achieved. Some students need more time- sometimes you see it in their faces. I give them a break and then I make a wanted repetition.” (SE1)* *“I ask the students to ask questions at any time and I try to get them all on the same track.” (SE2)* *“When I take them to the ward to see a patient and when they are allowed to wear their white coat, you see a glimpse in their eyes. I feel a great responsibility, they seem to absorb all that I do.” (JE2)*
*2. Disruption of face-to-face teaching units* The lack of face-to-face teachings endorsed the feeling of helplessness and incompetence through a decreased interaction with the lecturer and peers	*2.1 Increase of gaps in knowledge* Through the lack of face-to-face teachings gaps in knowledge cannot be clarified and the sense of incompetence grows; the connections of the learning contents are not easily seen, resulting in feelings of incompetence and overload *2.2 Decrease of class´ structure* Learning content and its load is less structured and the students feel incompetence and overload *2.3 Less confirmation of the learning cycle* Confirmation of the learned the confirmation of the learning process is reduced and the sense of incompetence increases; increasing feeling of deprivation	In SDT, amotivation is defined as utmost state of motivational deficit. Amotivation is often defined and characterized by feelings of alienation and helplessness. The growing feeling of incompetence induced by the disruption of face-to-face teachings increased the feeling of helplessness and resulted in amotivation. Ability beliefs vanish and nurture amotivation. Characteristics of the task (online lessons) were not stimulating and hence no high-quality experience in terms of knowledge gain took place. The result is enhanced amotivation. Students are impacted by adequate transmittance of information to accelerate the learning cycle- this results in competence support. The lack of face-to-face teachings impaired this acceleration, hence decreased the feeling of competence and resulted in amotivation.
***Sample statements for theme 2.***	*“I was so overwhelmed by the learning goals. Usually, I never looked at them but during the lockdown I felt that they are like a mountain I could never climb alone.” (S2)* *“It took me days to repeat and repeat things and I was not aware that I simply did not understand it properly” (S2)* *“The educators always gave us the feeling they will guide us through the learning process. Webex did not convey that. We could not really have a true interaction. I felt lost somehow and sometimes panicked even”. (S6)*	*“When I held my classes online, I lost the connection to my students. I knew some of them from prior classes and they knew me. The Interaction was not the same anymore. Usually, I considered myself as their navigational system through the semester. During the lockdown, I saw my students often being desperate but still not participating.” (SE1)* *“After the semester, I can tell you about how many students will have which kind of test results. I know them and I see how their learning progresses. During the lockdown I could not make any statement concerning any student.” (SE3)* *“It was frustrating often. I tried to talk to the students to see what else I could explain in order to help them with their learning, but it seemed as if they themselves desperately locked up” (JE1)*
*3. Disruption of patient contact* Patient interaction as a process facilitating the identification with the job (wanting to help patients) and hereby enhancing the feeling of competence and wish to learn	*3.1 Interaction with patients enhances the wish to help* *3.2 interaction with patients endorses the identification with being a doctor* Interaction with patients enhances the feeling of competence; the importance of the studies was lost in the students´ sight	Patient interaction functions as a catalysator in the identification process (being a doctor) and internalizing the task. The locus of causality for studying is seen from within, the more identification with being a doctor settles in. The subtype of amotivation, value placed on the task, decreases while identification decreases. The value of the task (being a doctor, hence studying) devalues. Values by implication, influence the perceived desirability of a task and hereby influence the organization of personal goals. All the mentioned was affected by the disrupted patient contact. Patients contact furthermore fosters the feeling of autonomy (I will be a doctor) which would facilitate the adoption of studying as an internal behavior. Here again the disruption led to a decrease of this internalization process.
***Sample statements for theme 3.***	*“When I went to bedside teaching, it was always so motivating. I knew why I study medicine and I knew why I want to learn in order to be able to help”. (S3)* *“Any time I had contact with a patient, my wish grew to be completely in charge for my own patients after medschool”. (S4)*	*“One does not need lots of effort to have a good bedside teaching. The students always are so motivated when they are in their future roles”. (SE1)* *“It is sometimes very adorable how students slip into their future roles. They love wearing the white coat and “playing” doctor” (SE3)* *“When we have seminars on a topic and then the next day go to ward to apply the gathered knowledge, the students are always so happy and eager” (JE1)*
*4. Disruption of daily university structure* Structure of university as an influencing factor of the daily life and individuals´ (students) feelings	*4.1 Decreased feeling of security by disruption of university structure* The structure given by the university (mandatory classes etc.) provides a feeling of security; the structure given by the university is something reliable and was absent during lockdown, resulting in increased feelings of helplessness *4.2 Decreased daily routine* The university structures the daily routine and the sudden absence results in insecurity *4.3 Decreased sense of freedom* Being a student and attending university creates a sense of freedom *4.4 Increased devaluation of university* classroom teaching and studying seemed more and more irrelevant	The role of the environment per se matters in terms of supporting the basic psychological needs. The structure of university endorses the sense of effort beliefs in relation to their time management (go to university, study afterwards and it will work out well). This feeling of reliability vanished by the closure and resulted in a feeling of alienation and helplessness- hence amotivation increased. The loss of structure increased the levels of perceived stress– resulting in a learned helplessness. The task (studying medicine- in terms of going to university) becomes a less integral part of students´ life and hereby its importance decreases- resulting in amotivation for studying medicine. The activity (studying medicine) becomes less self-expressed. Students´ autonomy vanished as due to the closure of university their own learning strategies were not fulfilled. Students did not feel to act out of free choice (I go to the library when I want, I don’t attend a non-mandatory lecture etc.). Hence the locus of causality moved to extrinsic regulation.
***Sample statements for theme 4.***	*“Prior to the lockdown it was easy. We had our schedule, and all other activities were planned around that.” (S1)* *“All of our daily routine was based on the structure given by the universities- it is different for us who go to medschool than other students- suddenly all was gone.” (S2)* *“It was such a good feeling to be a student, going to university- it felt so intellectual. After a while I lost the sense of studying- it was an awkward feeling.” (S2)*	*“When they start to go to medical school, they are still like pupils and their daily structure does not change actually. The Deans´office let them know when to attend which class. Although they feel free, they still are in schedule boundaries” (SE1)* *“Suddenly they lost their gathering place and they told me, without that place, why attend online classes” (SE1)* *“Some of the students told me they are feeling depressed. Mainly because university was their mainstay in life which defined so many things for them”. (JE2)*
*5. Social (peer) isolation through closure of universities* Closure of universities led to social isolation, enhancing insecurities and the feeling of incompetence Social (peer) isolation through closure of universities	*5.1 Increased feelings of alienation* Students were suddenly “all alone” and with reduced contact to their peers; feelings of deprivation increased; lack of contact with peers and lack of learning groups decreased the actual meaning of studying *5.2 Decreased role identification* The meaning of being a medical student was not seen *5.3 Decreased peer stress to endorse learning* Social pressure as a positive factor to endorse learning *5.4 Decreased perceived perspectives* Lack of perspectives; the importance of the task was not seen anymore (the why to study)	The broader social context in which the student is situated influence academic attitudes and behaviors and therefore amotivation. Relationships with others are a basis to fulfill individuals psychological needs. Either through needs that are satisfied or during an interaction where bilaterally needs are satisfied. These relationships (in this context mainly with peers) were disrupted by pandemic circumstances. Effort believes are stronger in communities and learning groups a.e enhance the feeling that the needed strategies are to master the task (studying medicine). Interaction with peers enhances the importance and hence the value of studying- here amotivation stemmed from devaluing the task due to deficits in interaction and lonely coping. The interaction with peers automatically centered university as an integral part of students´ lives- this vanished centering led to increased amotivation. The closure of universities led to frustration of students, mainly because of the lack of practice of practical skills- automatically their environment conveyed negative information about university (as listed in the evaluations). This led to devaluing university and hence studying medicine and hereby amotivation grew. The feeling of (knowledge) competence vanished as confirmation by peers was not possible as usual (a.e learning groups). The students did not have the chance to experience a supporting network and the feeling of competence, relatedness and autonomy decreased.
***Sample statements for theme 5.***	*“During the orientation phase (first week of university), most of the learning groups are formed. We go through the learning phases together, we talk about our fears, we set goals and try to achieve them together- we even have lunch and coffee together. During the pandemic all was gone and we could not even meet in private. Online meetings were no substitute. It was a bad feeling.” (S1)* *“I often talked to my peers about what we would like to become- in sense of which specialty. We evaluated our learning and helped each other. Suddenly all this interaction was gone” (S5)* *“It is being with friends but also a friendly competition is motivating” (S6)* *“The feeling: we are in this together was gone”. (S7)*	*“They are like small formations on the campus, and they share a lot of things to facilitate the stressful learning phases. I imagine it to be very hard for them to be all alone at once”. (SE2)* *“I remember from my student time, that my learning groups were like family. They stressed also because if we did not learn like we set our goals, we would be the “looser”. (JE2)*

Note: The terms “ability beliefs, effort beliefs, characteristics of the task, value placed on the task” are complementary aspects of amotivation, sharing a common core and covary with one another [36]. The focus groups were conducted until saturation was reached. Therefore, all the statements reflect the whole group/opinion or statement of each participant. Abbreviations: SDT = Self-determination Theory; S = Student; SE = Senior educator; JE = Junior educator.

## Discussion

In our mixed method inter-cohort study, we assessed the motivational changes that took place during the pandemic in medical students, explored and identified determinants and confounders of students´ motivation systematically and specifically caused by pandemic circumstances. Our results provide several implications and explanations regarding the affective dimension of learning; a crucial dimension which has often been neglected in the context of medical education [17].

Our hypothesis, that during the lockdown autonomous motivation towards studying medicine had grown, was not confirmed. Our hypothesis was grounded on the assumption, that due to the lockdowns and restricted access to university, students` desire to participate in the classes and to study medicine had grown. Hereby the locus of causality (the why of goal pursuit) had shifted more towards inside, resulting in enhanced autonomous regulation [8, 37]. Contrary to our hypothesis, after the relaunch of university, students reported even inferior forms of motivation, characterized by lower levels of self-determined regulation [38]. The explorative qualitative analysis revealed five core themes (determinants) to explain how motivation of medical students is influenced and how the pandemic circumstances had affected students´ motivation. These determinants share the common core of lacking social interaction and support. Our results can also be embedded in the SDT: According to SDT, it is the relationship with others that enables individuals to fulfill their psychological needs (autonomy, competence and relatedness) [36, 38]. Our results demonstrate that the pandemic related circumstances (closure of universities with lacking social interaction and support) hampered the satisfaction of the basic psychological needs. The more these needs are satisfied, superior forms of motivation and therefore higher levels of self-determination are present [8, 39]. According to the Cognitive Evaluation Theory (CET) [39], a sub theory of the SDT, social context has three main dimensions. Autonomy support, competence support and interpersonal affiliations (relatedness) [39]. Herein lies the explanation for the increased levels of extrinsic regulation which we found: During the pandemic lockdown social support was lacking. Hence, students had no opportunity to build stable relationships with their social context and authority figures (lecturers), resulting in decreased feelings of affiliation [36, 40]. Students´ autonomy was also not supported. The structure of the classes was completely given with no possibility for the students to engage in self-chosen further activities. These circumstances decreased the feeling to act out of free choice and hereby the locus of causality moved towards outward and identification with the task decreased [8, 41, 42]. The feeling of competence was also not supported. On the one hand due to the lacking feedback of peers (learning groups etc.) and on the other hand due to the reduced directive feedback of the lecturers during online classes.

Next to enhanced levels of extrinsic regulation, the students also reported significant higher levels of amotivation than the pre-pandemic cohort. SDT traditionally defines amotivation as a state in which motivation is lacking. The individual perceives the reason for its action from outside (alienation) and feelings of disintegration from the action (learned helplessness) will result [39, 43]. In a more expanded sight on amotivation Legault et al. explain in a multi-dimensional construct, that academic amotivation occurs for four different classes of reasons, hence amotivation has four subtypes: academic amotivation based on ability beliefs, effort beliefs, characteristics of the task, and value placed on the task [36]. These subtypes of motivation are conceptualized as complementary and share a common reason and are still connected to alienation and helplessness [36]. The results of our qualitative analysis reconfirm this construct of amotivation in the context of medical education. Due to the disruption of the face-to-face teachings and reduced direct feedback, the learning cycle was not promoted as usual [44]. Students experienced decreased beliefs about their abilities to apply necessary strategies to accomplish the learning contents [45]. Failure became more present in students´ perceptions, as the feeling of self-efficacy was fading. Consequently, the students perceived their efforts as insufficient [46, 47].

During the lockdown, the feelings of deprivation of students increased and based on the end-term evaluations of the Faculty of Medicine Hamburg, they did not value university positively- or better to state- the online version of university in that period of time. The devaluation of the task is an important variable which led to amotivation [48], as it led to vanished inner acceptance of the task, studying moved away from being an integral part of their lives and hence studying was no longer a way of self-expression. The identification with the task moved from inside to outside, resulting in higher levels of controlled regulation- or- amotivation.

Since the pandemic, research on the field of medical students´ motivation has tremendously increased. Majority of the studies focus on the challenges and opportunities of distance teaching [49, 50] and its perception [51, 52] with regard to medical students´ motivation.

However, no published study sought to explore the affective (motivational) dimension of learning as a whole and embed the results within a broader context of motivational theories. Herein lies a strength of our study- we analyzed motivation in a broader context, stretching beyond the educational domain only. Next to the explanations by the SDT, we open a new scope to interpret the course and change of medical students´ motivation, including aspects of pandemic related restrictions. Hereby, several implications for the educational practice derive.

Although remote learning and online teachings have gained popularity [53, 54] by providing facilitated access to learning materials [55], their disadvantages also merit consideration [53, 56]. Our results also support the negative effects of online classes as a substitute on medical students´ motivation, mainly based on the importance of a given structure, peer-contact and interaction. Prior to the pandemic, research from the general- and non-pandemic field of distance teaching has already provided a great insight on demotivational factors influencing learners. Amongst others, anxiety and experience with failure, a lack of self-learning organization, lack of peer-environment (isolated learning), a lack of simultaneous communication, technical problems, and detrimental teachers´ behaviors have been described [57–62]. Although our findings are in accordance with- and confirm the reported factors, we still believe that the pandemic effects on medical students´ motivation need to be analyzed separately and in a broader context. The previous constructs of demotivation are not completely transferable to the pandemic circumstances and its consequences for future medical doctors. As an example, if we consider the demotivational construct as described by Dörnyei and colleagues, sources of demotivation are of external and internal origin. The demotivation is mostly externally triggered and then internalized [63]. In the context of our study, the forced disruption of face-to-face teaching might be considered as the external demotivator. Alas, the main difference of this construct and the pandemic circumstances lies within the unchangeable nature of latter and not being a chosen circumstance like distance learning. Therefore, the motivational effects created by the pandemic are not limited to learning and teaching activities but include fears and expectations. It is not only the milieu in which the students had to learn, but the lack of identification with future responsibilities and the fear of not acquiring necessary skills and hereby harming patients. The convergence of the globally considered events, embedded in the circumstances of the medical universities, resulted in the changes in behavioral regulation (motivation) we found.

Furthermore, we found patient contact to be important to enhance autonomy, competence and to facilitate the internalization of the task and the identification process with being a doctor. Here an important implication for further curriculum developments and amendments derives: Patient contact should be supported and facilitated during medical school. A teaching method which already exists in medical curricula and known as “bedside teaching”, [64] should be further evolved and tailored to the autonomy needs of medical students. The patient contact needs to foster the sense of being a doctor in students, by allowing them to act more autonomously and self-determined. Certainly, a paradigm shift, as so far, the focus of bedside teaching was content driven, namely conveying practical and communication skills [64].

Our findings emphasize the crucial role of the medical teacher in the development of ability conceptions, as well in the identification process of medical students. The medical teacher functions as a role model, fostering the inner perceived reason of why the activity (studying medicine, study efforts) is pursued. The relationship with this authority figure nourishes the identification and the ability conceptions of students. Furthermore, by providing feedback and conveying the teaching content in a sufficient and interesting manner, the teacher is a catalyst for the learning cycle. So far, in medical education specifically, recommendations have been published on how to engage in autonomy supportive teaching behaviors [65]. Our results supplement these recommendations and mainly emphasize the impact of the medical teacher as a role model. As medical teachers are traditionally clinicians or researchers [66], medical faculties should expand their offerings for medical teachers the be further educated in sense of pedagogical and psychological skills (a.e train the trainer programs).

Some limitations of our study merit consideration. First, we assessed motivation of 4th year students during their anaesthesia module, which might question the generalizability of our findings. This can be overruled, because the students were also scheduled for other teaching units of other specialties during the study period. Furthermore, we assessed the situational motivation, which is detached from the contextual level of motivation [15] and we specifically asked for the reason why the students studied medicine.

A further limitation of our study is the inter-cohort design. We compared motivation of different cohorts at different times, disregarding intra-cohort variances. Nevertheless, the pandemic occurred unexpectedly and therefore, a prospective design was not possible. Furthermore, we tried to reduce confounding factors as we compared students who attended the same class at the same season.

Although our medical teachers put their maximum of effort inside the design of their online teachings and quality standards were defined, results of other universities, with other online classes might have led to other changes in students´ motivation. Therefore, it is questionable to generalize our quantitative results as “the pandemic effects” on students´ motivation. However, it should be accentuated that first, our qualitative analysis confirmed the results of our quantitative analysis. Secondly, we explored determinants of student motivation independently from the quantitative data determinants of student motivation. These determinants can be considered as unpaired and valid results. Due to the design of our qualitative analysis, our claims are critically evaluated and therefore, data rigor is given. Herein lies a further strength of our study, we applied a threefold analytic strategy. With data from the quantitative analysis (data-driven) we assessed the current state, confirmed the results with a qualitative analysis (explanation-driven) and then also embedded the results within the leading theory of motivation, SDT (theory-driven).

## Conclusion

Our findings are in accordance with the SDT and can be transferred to any medical educational field. Next to educational strategies, stakeholders of medical education should value the motivational dimension of learning and enhance consciousness among medical educators on the impact that students´ motivation has on several outcomes. We undermine the crucial role of social support, patient contact and the interaction with the medical educator to enhance the feelings of autonomy, competence and relatedness. Students need to be strengthened in their beliefs about their abilities, their efforts as well as in their beliefs about the value of the task. All this will result in an increased identification with the task and less detrimental outcomes.

## Data Availability

Data generated in the study is displayed in the manuscript. More details are available from the corresponding author on reasonable request.
